# New Sequence Types of *Vibrio parahaemolyticus* Isolated from a Malaysian Aquaculture Pond, as Revealed by Whole-Genome Sequencing

**DOI:** 10.1128/genomeA.00302-17

**Published:** 2017-05-11

**Authors:** Soon Man Foo, Wilhelm Wei Han Eng, Yin Peng Lee, Kimberly Gui, Han Ming Gan

**Affiliations:** a School of Science, Monash University Malaysia, Petaling Jaya, Malaysia; b Genomics Facility, Tropical Medicine and Biology Platform, Monash University Malaysia, Petaling Jaya, Malaysia

## Abstract

The acquisition of *Photorhabdus* insect-related (Pir) toxin-like genes in *Vibrio parahaemolyticus* has been linked to hepatopancreatic necrosis disease in shrimp. We report the whole-genome sequences of genetically virulent and avirulent *V. parahaemolyticus* isolated from a Malaysian aquaculture pond and show that they represent previously unreported sequence types of *V. parahaemolyticus*.

## GENOME ANNOUNCEMENT

*Vibrio parahaemolyticus* is a marine Gram-negative bacterium (1) that has been occasionally associated with acute hepatopancreatic necrosis disease (AHPND) in the white leg shrimp, *Litopenaeus vannamei*, resulting in severe economic losses in shrimp production in Southeast Asian countries (2). *Photorhabdus* insect-related (Pir) toxin-like genes have been recently identified in various AHPND-causing *V. parahaemolyticus* strains and these genes (*pirA*- and *pirB*-like) were shown to be the primary virulence factor in these strains (3).

Five* V. parahaemolyticus* strains (MVP1, MVP2, MVP4, MVP6, and MVP9) were isolated from a shrimp pond that was tested positive for *V. parahaemolyticus* harboring the Pir genes. Genomic DNA was extracted from a 2-day-old marine nutrient agar culture (ATCC Medium 8) using the Solokov method (4). Library preparation was performed using the NexteraXT DNA library preparation kit (Illumina, San Diego, CA) according to the manufacturer’s instructions and sequenced on a MiSeq desktop sequencer (2 × 75-bp and 2 × 250-bp configurations) located at the Monash University Malaysia Genomics Facility.

Nextera adapter trimming was performed using Trimmomatic version 0.32 (5) and the filtered paired-end reads were assembled using SPAdes version 3.8.1 (6). After the removal of short (<300 bp) and/or low-coverage (<2×) contigs, *in silico* scaffolding and gap closing were performed using SSPACE version 2.1 (7) and Gapfiller version 1.10 (8), respectively. To confirm the identity of the isolated strains as *V. parahaemolyticus*, Jspecies version 1.2 (9) was used to calculate the average nucleotide identity of strains MVP1, -2, -4, -6, and -9 in comparison to the whole genome of *V. parahaemolyticus* DSM 10027^T^. Subsequently, gene prediction was performed using Prodigal version 2.6 (10) and searched against the multilocus sequence typing (MLST) locus database (http://www.mlst.net/) to infer the sequence type of each sequenced strain based on their genetic similarity to seven housekeeping genes, namely, *pyrC*,* gyrB*,* recA*,* dnaE*,* tnaA*, *pntA*, and *dtdS*. The identification of the Pir genes was performed via a local BlastN search against *pirA* (GenBank accession no. AIL49948.1) and *pirB* (GenBank accession no. AIL49949.1).

A summary of the assembly statistics for the genomes of all isolates is available in Table 1. All five strains exhibited more than 95% average nucleotide identity (ANI) to the type strain of *V. parahaemolyticus*. Based on the lack of 100% sequence identity to seven housekeeping genes, new MLST sequence types of *V. parahaemolyticus* were identified in this study (Table 1). Subsequent blastN searches showed that strains MVP1, MVP2, and MVP6 contain the identical nucleotide sequence for all seven housekeeping genes (Table 1, data not shown), thus classifying them as the same sequence type. In addition to sharing the identical sequence type, these 3 strains also harbor the Pir toxin genes. On the contrary, strains MVP4 and MVP9 belong to two different sequence types and do not harbor the Pir toxin genes, suggesting a potential association between the sequence types and the presence of Pir toxin genes in *V. parahaemolyticus*.

**TABLE 1:** Accession numbers and genetic analyses of *V. parahaemolyticus* strains reported in this study

Strain	Accession no.	Genome size (bp)	*N*_50_ (bp)	No. of contigs	*pyrC^a^*	*gyrB^a^*	*recA^a^*	*dnaE^a^*	*tnaA^a^*	*pntA^a^*	*dtdS^a^*	Pir^b^
MVP1	MQMQ01000000	5,230,330	60,033	172	303	143^c^ (591/592)	218	308^c^ (556/557)	26	30	355^c^ (457/458)	+
MVP2	MSBY01000000	5,275,177	129,821	89	303	143^c^ (591/592)	218	308^c^ (556/557)	26	30	355^c^ (457/458)	+
MVP4	MSBZ01000000	5,270,749	94,277	122	27	141	31^c^ (728/729)	110^c^ (556/557)	26	18	232	−
MVP6	MSCA01000000	5,195,990	47,800	190	303	143^c^ (591/592)	218	308^c^ (556/557)	26	30	355^c^ (457/458)	+
MVP9	MSCB01000000	4,967,664	87,871	115	54^c^ (492/493)	144	116	28	61	26	252	−

a Numerical values indicate the MLST allele for the respective genes.

b +, presence of both *pirA* and *pirB* genes; −, absence of both *pirA* and *pirB* genes.

c Closest allele hit, with values in parentheses indicating the number of positions over the length of the gene fragment where all of the bases at that position are identical.

### Accession number(s).

This whole-genome shotgun project has been deposited in DDBJ/ENA/GenBank under the accession numbers listed in Table 1. The versions described in this paper are the first versions.
